# Projections of Brodmann Area 6 to the Pyramidal Tract in Humans: Quantifications Using High Angular Resolution Data

**DOI:** 10.3389/fncir.2019.00062

**Published:** 2019-09-26

**Authors:** Zhen-Ming Wang, Yi Shan, Miao Zhang, Peng-Hu Wei, Qiong-Ge Li, Ya-Yan Yin, Jie Lu

**Affiliations:** ^1^Department of Radiology, Xuanwu Hospital, Capital Medical University, Beijing, China; ^2^Beijing Key Laboratory of Magnetic Resonance Imaging and Brain Informatics, Beijing, China; ^3^Department of Neurosurgery, Xuanwu Hospital, Capital Medical University, Beijing, China; ^4^Department of Nuclear Medicine, Xuanwu Hospital, Capital Medical University, Beijing, China

**Keywords:** supplementary motor area, dorsal premotor area, high angular resolution diffusion imaging, pyramidal tract, human connectome project

## Abstract

Primate studies indicate that the pyramidal tract (PyT) could originate from Brodmann area (BA) 6. However, in humans, the accurate origin of PyT from BA 6 is still uncertain owing to difficulties in visualizing anatomical features such as the fanning shape at the corona radiata and multiple crossings at the semioval centrum. High angular-resolution diffusion imaging (HARDI) could reliably replicate these anatomical features. We explored the origin of the human PyT from BA 6 using HARDI. With HARDI data of 30 adults from the Massachusetts General Hospital-Human Connectome Project (MGH-HCP) database and the HCP 1021 template (average of 1021 HCP diffusion data), we visualized the PyT at the 30-averaged group level and the 1021 large-sample level and validated the observations in each of the individuals. Endpoints of the fibers within each subregion were quantified. PyT fibers originating from the BA 6 were consistently visualized in all images. Specifically, the bilateral supplementary motor area (SMA) and dorsal premotor area (dPMA) were consistently found to contribute to the PyT. PyT fibers from BA 6 and those from BA 4 exhibited a twisting topology. The PyT contains fibers originating from the SMA and dPMA in BA 6. Infarction of these regions or aging would result in incomplete provision of information to the PyT and concomitant decreases in motor planning and coordination abilities.

## Introduction

The pyramidal tract (PyT) is a longitudinal pathway that delivers information regarding voluntary motor behavior. It is constituted of the corticospinal tract and the corticobulbar tract (Rea, 2015). According to experiments using non-human primates, beside the primary motor area, the PyT could still originate from Brodmann area (BA) 6. Specifically, in the BA 6, the PyT predominately derives from the supplementary motor area (SMA), dorsal premotor area (dPMA), and cingulate cortex (Dum and Strick, 1991; Galea and Darian-Smith, 1994). In humans, diffusion tensor imaging (DTI) has been introduced to visualize the PyT (Kumar et al., 2009; Zolal et al., 2012; George et al., 2014). As the DTI technique cannot discriminate fanning or crossing fibers, or flipped angles (Fernandez-Miranda et al., 2012), it cannot decode either the fanning shape of the corona radiate (Rea, 2015) or the multiple crossing fibers at the semioval centrum (Fernandez-Miranda et al., 2012). A failure to visualize these anatomical features means that the origin of the PyT from regions other than BA 4 (e.g., fibers from BA 6) remains unknown in humans (Archer et al., 2018; Chenot et al., 2019).

With the introduction of multi-band techniques (Moeller et al., 2010), many diffusion directions and multiple *b*-values became available. Consequently, high angular resolution acquisition techniques, such as diffusion spectrum imaging (DSI) and high angular-resolution diffusion imaging (HARDI) have been developed to solve the aforementioned shortcomings of DTI. Besides, high angular resolution acquisition approaches could determine both the origin and termination with accuracy compared with traditional DTI, specifically, the accurate termination could even outline the shape of cortical gyrus (Fernandez-Miranda et al., 2012). In addition to the scanning technique, quantitative anisotropy (QA)-based deterministic tracking methods (Yeh et al., 2013) were also developed with specific focus on high angular-resolution data. This algorithm can interpolate multiple orientations directly to a single voxel by considering the anisotropy of different b-vectors and the trajectory, and can reliably present anatomical features such as fanning, crossing, and angular-flipping fibers, and fiber terminations (Fernandez-Miranda et al., 2012; Yoshino et al., 2016; Wei et al., 2017). In addition to the high angular resolution techniques, high magnetic gradient strength could shorten the diffusion encoding time enormously and decrease the signal loss owing to T2 decay (Fan Q. et al., 2016). The advantages of the high magnetic gradient have been validated by the comparison of tractographies under different gradients of 300 mT/m, 80 mT/m and 40 mT/m (Chamberland et al., 2018). Thus, constraints that previously hindered explorations of the termination of the PyT have largely been eliminated. Accordingly, in the present study, we explored projections from BA 6 to the PyT using these high angular-resolution diffusion techniques and high magnetic gradient data.

## Materials and Methods

In the present study, 30 groups of the Massachusetts General Hospital-Human Connectome Project (MGH-HCP) individual diffusion data from the HCP were used^1^. This dataset was acquired at the MGH, using the Siemens 3T connectome scanner, which has 300 mT/m maximum gradient strength (McNab et al., 2013; Setsompop et al., 2013). High gradient strength can improve the angular resolution of the diffusion image (Fan Q. et al., 2016). First, the 30 individual datasets were averaged to the same Montreal Neurological Institute (MNI) space to create a 30-subjects-averaged template which was created by ourselves. Fiber tracking was performed for this template. Then, we performed the fiber tracking using another open source template^2^, which consisted of 1,021 healthy young adults from the HCP (Q1-Q4, 2017) and did not need data preprocessing. Finally, fiber tracking was performed for the 30 participants at the individual level in individual space to validate the findings from the two templates. For each type of data or template, the fiber tracking process was divided into two stages. In the first stage, we performed fiber tracking to test whether the fanning shape of the corona radiata and multiple crossings could be reliably presented, as this constituted the basis of exploring the origin of the PyT. In the second stage, we quantified the effective connectivity of the projections from BA6 and its relationship with BA4. Thus, these two stages were performed at the individual level, group level (30-subjects-averaged template), and the large population level (HCP 1021 template). Parameters of the scanning protocol are available at: http://protocols.humanconnectome.org/HCP/3T/imaging-protocols.html and http://protocols.humanconnectome.org/HCP/MGH/. The study was reviewed and approved by Xuanwu Ethical Committee.

### Data Preprocessing

We used the open access software DSI-studio^3^ to analyze the data. First, we reconstructed the spin distribution function (SDF) of each participant and simultaneously warped the SDF to the MNI space, using the q-space diffeomorphic reconstruction method (Yeh and Tseng, 2011). A value of 1.1 was used as the diffusion sampling length ratio. All these 30 groups of SDFs were averaged to the same MNI space to create the 30-subjects-averaged template. Then fiber tracking was performed with this template.

For the preprocessing in the individual space, the SDF was reconstructed using the generalized q-sampling imaging method. The following parameters were used: diffusion sampling length ratio 1:1, 20-fold SDF tessellation, 10 resolved fibers. The SDF was used to conduct further fiber tracking in the individual space to validate the findings.

### Fiber Tracking

Fiber tracking was performed for the 30 individuals, the 30-subjects-averaged template, and the HCP 1021 template. To track the multiple crossing fibers, we first visualized the left-right oriented fibers that extended from the corpus callosum by drawing a region of interest (ROI) at the corpus callosum and lateral side of the semioval centrum in sagittal slices. The frontal-posterior oriented fibers of the arcuate fasciculus were visualized by setting the superior temporal gyrus and premotor cortex as ROIs. The superior temporal gyrus and the premotor cortex were defined by the non-liner registration of the automatic anatomical labeling (AAL) atlas and Brodmann atlas, respectively, to the individual space within the DSI-studio software. The superior-inferior oriented fibers of the pyramidal tract were visualized as follows: to track the potential PyT fibers issuing from BA 4 (PyT4) and 6 (PyT6), we introduced the Brainnetome Atlas^4^, which is a structural connectome based template (Fan L. et al., 2016) in which BA 6 was parceled into the caudal dorsolateral region (A6cdl), caudal ventrolateral region (A6cvl), dorsolateral region (A6dl, or dPMA), ventrolateral region (A6vl), and medial region (A6m, or SMA), while BA 4 was parceled into the head and face region (A4hf), upper-limb region (A4ul), trunk region (A4t), tongue and larynx region (A4tl), and lower limb region (A4ll). During fiber tracking, these cortical regions were used as seed regions. Additionally, ROIs in the white matter included the cerebral peduncle (imported from the JHU white-matter tractography atlas, which is embedded in the DSI-studio software) and the pyramid (drawn by a neurologist with 10 years of experience). In each round of fiber tracking, one cortical seed region was paired simultaneously with the cerebral peduncle and pyramid to visualize the PyT.

Most of the ROIs within the present study were imported to the native space by non-linear registration using open access atlas, the whole processes were performed without subjective manipulations. For the pyramid, it is an obvious anatomical structure which was bordered medially by the anterior median fissure and laterally by the anterolateral sulcus. Therefore, the repeatability of defining the regions was also guaranteed.

The QA threshold was set at the optimal threshold such that the orientation distribution signal best fit the brain tissue in the SDF map, with the least amount of the orientation signal falling outside the pial boundary or into the ventricle. Here, values of the QA thresholds ranged from 0.05 to 0.1 for the individual data, while the value was 0.15 for the 30-subjects-averaged template and 0.1 for the HCP 1021 template. Other tracking parameters were as follows: angular threshold = 90, step size = 0.5, smoothing = 0.8, min length = 5.0 mm, max length = 300.0 mm, trilinear algorithm, and streamlined. The tracking process was set to terminate if the seed number reached 50,000.

### Connectome Evaluation

To evaluate whether a given region within BA 6 is an effective region that constituted the PyT, we defined the effective index (EI) as the number of endpoints that fell within the region divided by the number of voxels in the region; this reflects the average fiber distribution within the region. Calculation of the EI was performed for the 30-subjects-averaged template and HCP 1021 template, to estimate the composition of the PyT at the group or population level. Here, 0.5 was chosen as the EI threshold; that is, if more than half of the total voxels within a certain region had at least one endpoint, we considered this region to reliably contribute fibers to the PyT. Origin analysis (EI), shape, and course of the fiber tracts were observed for the 30-subjects-averaged template and HCP 1021 template, and verified in 30 of the MGH-HCP individuals.

## Results

In the 30-subjects-averaged template, HCP 1021 template, and all 30 individuals, fibers of BA 6 were consistently observed to contribute to the PyT. Additionally, the trajectory and shape of the PyT were also visualized at individual, group, and population levels.

### Fanning Shape and Triple Cross of the PyT

**Figure 1:**
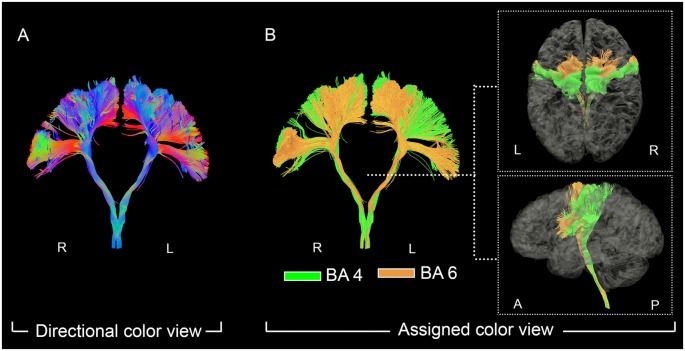
The fanning shape of pyramidal tract (PyT) visualized in individual level. On the left panel **(A)**, the fanning shape of PyT was visualized in the directional view. On the right panel **(B)**, PyT4 and PyT6 are shown in green and orange, respectively, together with top and lateral views.

**Figure 2:**
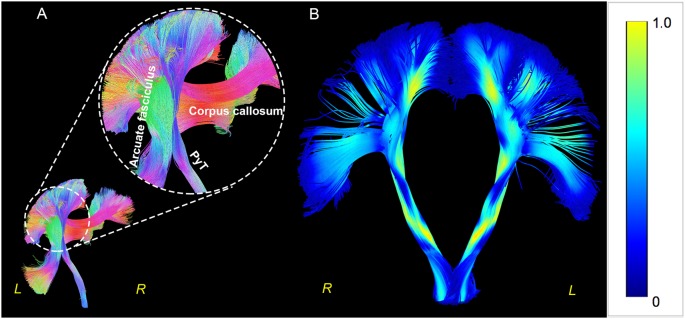
Triple crossings and fanning shape in Human Connectome Project (HCP) 1021. Triple crossings at the semioval centrum, PyT, corpus callosum, and arcuate fasciculus are shown in the left panel **(A)**. The fanning shape is shown in the right panel **(B)**, with color-code indicating the quantitative anisotropy (QA) value. The QA values were observed to be higher in white matters and lower within the gray matters.

We first checked whether an inability to reconstruct the fanning shape and triple cross, which would prevent visualization of the endpoints of the PyT, were present for the templates and individual-level data. Fanning shapes were present in most of the data (Figures 1, 2). Additionally, triple crossings were visualized at the semioval centrum, which consisted of fibers of the corpus callosum in a left-right direction; arcuate fasciculus in the anterior-posterior direction; and PyT in the superior-inferior direction (Figure 2).

### Quantitative Evaluation of PyT Distribution in BA 6

**Figure 3:**
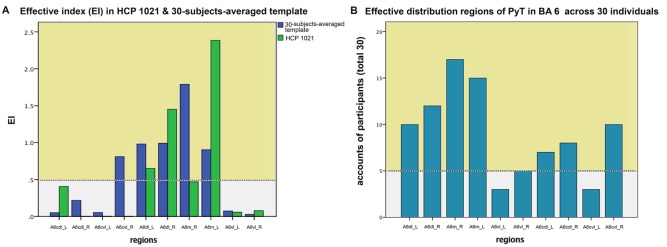
Effective index (EI) in HCP 1021, 30-subjects-averaged template and across 30 individuals. On the left panel **(A)**, subregions where EI was larger than 0.5 were bilateral A6dl and A6m, as well as the right A6cvl for the 30-subjects-averaged template. For the HCP template, these areas were bilateral A6dl and A6m. On the right panel **(B)**, bilateral A6dl and A6m, as well as the right A6cvl were the most frequently observed to be effectively contributed to the PyT across 30 individuals. Effective distributions are shown in yellow background.

**Figure 4:**
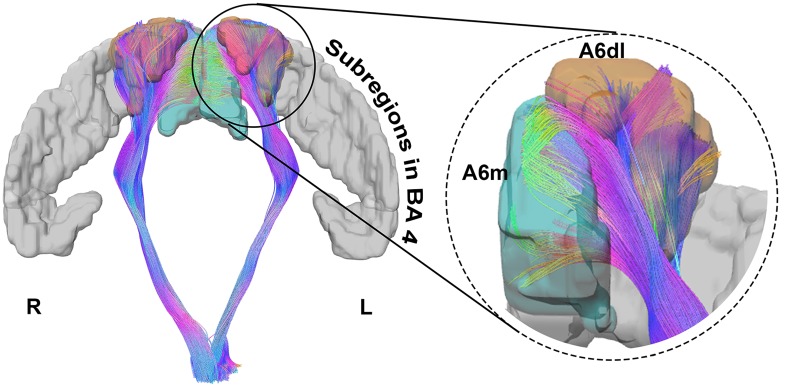
Details of the fiber tractography originating from A6dl and A6m in HCP 1021 template. The left side shows bilateral fibers that originated from A6dl and A6m, together with all subregions in Brodmann area (BA) 4 in gray. The right side shows details of fiber tractography origins from left A6dl and A6m. Left A6dl is shown in orange and left A6m in green.

For the 30-subjects-averaged template and HCP 1021 template, we calculated the EI according to subregions of BA 6 (Figure 3). For the former template, subregions where EI was larger than 0.5 were bilateral A6dl and A6m, as well as the right A6cvl. On the latter template, these areas were bilateral A6dl and A6m. Notably, subregions with consistent effective distributions between templates were bilateral A6dl and A6m. These findings were further verified in the individuals; we observed that the subregions of bilateral A6dl, A6m, and right A6cvl, had relatively high frequencies of being areas with effective distribution to the PyT (Figure 3). Details of the fiber tractography that originated from A6dl and A6m is shown in Figure 4.

### Twisting Relationship Between PyT6 and PyT4

**Figure 5:**
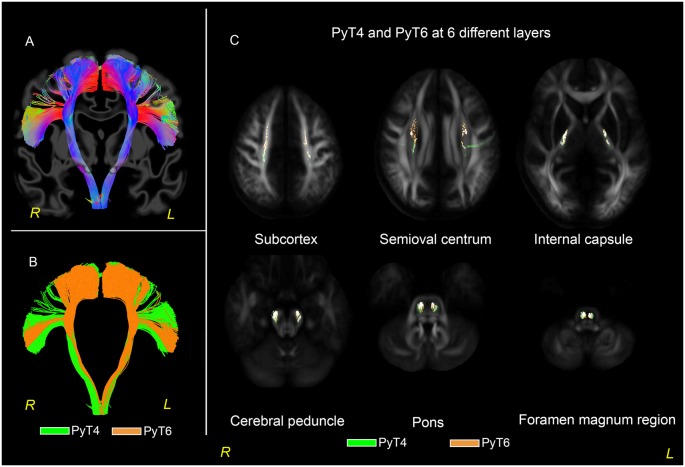
PyT6 and PyT4 in 30-subjects-averaged template. **(A)** Fibers distributed in a fanning shape ranged from the medial wall of the frontal lobe to the opercula region. **(B)** PyT4 and PyT6 are shown in green and orange, respectively. **(C)** PyT4 and PyT6 at layers of subcortex, semioval centrum, internal capsule, cerebral peduncle, pons, and foramen magnum region.

In both the 30-subjects-averaged template and HCP 1021 template (Figure 5), PyT6 was typically located anterior and medial to PyT4. Specifically, from the level of the cortex to the semioval centrum, PyT6 predominately coursed in front of PyT4. When descending into the internal capsule and the cerebral peduncle, PyT6 was located at the anteromedial side of PyT4. After further descending into the pons, PyT6 was located at the medial side of PyT4. At the level of the foramen magnum, PyT6 rotated to the medial posterior side of PyT4. These observations also applied to individual participants.

## Discussion

Using the large population data of the HCP and high magnet gradient HARDI data, we found the existence of PyT6 in humans. Specifically, A6dl, or the dPMA, and A6m, or the SMA, have consistently contributed to the PyT. To the best of our knowledge, there is a lack of extant literature that explores the distribution of fibers from BA 6 to the PyT in human beings.

Currently, most knowledge regarding the origin of the PyT was obtained from axonal tracing studies of non-human primates. These animal studies revealed that besides the primary motor cortex, the remaining projections originated from the SMA, arcuate premotor area, and caudal cingulate motor areas (Dum and Strick, 1991; Galea and Darian-Smith, 1994). Concerning the PyT in humans, although five decades ago postmortem observation of the human brain observed the PyT had origins other than the precentral gyrus (Jane et al., 1967), the definite origin of the PyT in humans remains to be defined (Archer et al., 2018; Chenot et al., 2019). Several DTI studies have explored the PyT (Kumar et al., 2009; Zolal et al., 2012; George et al., 2014); however, due to angular resolution limits, the fanning shape of the PyT and multiple crossings of the semioval centrum have been barriers to reliably evaluating the origin and course of the PyT. Additionally, most of these previous studies primarily focused on the precentral area. With respect to BA 6 projections to the PyT in humans, Seo and Jang (2013) investigated different diffusion indices of the SMA and dPMA, recognizing that the origin of the PyT at the SMA and dPMA was chosen by default in animal studies, rather than by defining explicitly which subregions project to the PyT (Seo and Jang, 2013). In a more recent study, Chenot et al. (2019) used DTI to create a PyT template. They observed the origin of the PyT as the premotor area; nevertheless, they also mentioned that the distribution to the PyT from the premotor area in humans remains to be defined (Chenot et al., 2019).

In the present study, the triple crossing fibers at the semioval centrum and the fanning shape of the PyT were visualized successfully, indicating that prior barriers that prevented visualization of the terminations had been removed. The QA values were observed to be slightly lower in the gray matter than in the white matter (Figure 2). This was due to white matter typically consists of axons in a certain direction, therefore, the diffusional movement of the water molecules are directive within the membrane (Alexander et al., 2007), and this resulted in a higher QA value. However, when approaching the gray matter, different layers of cytoarchitecture (Fatterpekar et al., 2002) typically decreased the water diffusion, thus, the QA value exhibited a reduction at regions of gray matter.

By using different subregions from the BN atlas that fully covered the entire BA 6, and by introducing EI as a quantitative evaluation of the effective distribution, we observed that the dPMA and SMA consistently contributed to the PyT; these observations are consistent with previous studies of non-human primates. According to the literature, the dPMA is primarily concerned with addressing external cues and adjusting movement plans (Paus, 2001; Hartwigsen et al., 2012). Additionally, the dPMA plays a role in the recovery of motor paresis after stroke (Watson et al., 1986; Seitz et al., 1998; Di Pino et al., 2014); injury to the dPMA can be accompanied by limb-kinetic apraxia (Freund and Hummelsheim, 1985; Jang and Seo, 2016). In contrast, the SMA is involved in self-initiated movements (Passingham et al., 2010), action monitoring (Bonini et al., 2014), and sequencing (Tanji, 2001). Infarction of the SMA can result in apraxia of the extremities (Marchetti and Della Sala, 1997; Chang and Chun, 2015). Watson et al. (1986) reported two patients with left mesial hemisphere infarctions that included the SMA who had bilateral apraxia for lower limb movements. Ito et al. (2013) reported a patient who was unable to move his left leg intentionally either by verbal command or by imitation after the SMA injury. Chang and Chun (2015) reported a patient with SMA infarction who presented lower limb apraxia and even noted that this apraxia might accompany disruption of the PyT. Therefore, this study might still have important clinical meanings.

Regarding organization of the PyT at different descending levels, we found that the PyT4 and PyT6 exhibited a twisting relationship from the level of cortex to the level of the medulla oblongata. The twisting organization of the PyT from the cortex to the internal capsule has been observed in previous studies (Park et al., 2008; Pan et al., 2012; Chenot et al., 2019), most of which focused on discussing the somatopology of the primary motor area in the internal capsule. Studies that explicitly assess the relationship between PyT6 and PyT4 are lacking.

There are limitations to the present study. First, it was previously reported that the PyT contains fibers from the cingulate cortex in non-human primates. However, in the present study, we did not find these projections after tracking all data carefully, even though the diffusion data we used in the present study is of high angular resolution. Thus, it remains to be elucidated whether the cingulate cortex is an origin of the PyT in humans. Next, in addition to the consistent regions of A6m and A6dl, other regions were variable and could not be defined meaningfully in the current study. Furthermore, there is only one modality and a lack of functional experiments exists in this study. Thus, the present study focused on discussing the structural basis of the PyT6. Future studies are also needed to explore the functional aspects of the fibers.

## Conclusion

Using HARDI images, our findings indicated that the PyT contains fibers originating from BA 6, most probably from the SMA and dPMA. Injury to the SMA and dPMA (e.g., infarction, aging) would result in incomplete receipt of information by the PyT, and further decrease motor planning and coordination abilities.

## Data Availability Statement

Publicly available datasets were analyzed in this study. The datasets analyzed for this study can be found at https://db.humanconnectome.org.

## Ethics Statement

This study was carried out with written informed consent from all subjects. All subjects gave written informed consent in accordance with the Declaration of Helsinki. The protocol was approved by the “Ethical committee of Xuanwu Hospital.”

## Author Contributions

Z-MW, P-HW and JL: conception and design. Z-MW, YS, MZ, Q-GL and Y-YY: development of methodology and data management. Z-MW: analysis and interpretation of data. Z-MW and JL: writing and/or revision of the manuscript. JL: study supervision.

## Conflict of Interest

The authors declare that the research was conducted in the absence of any commercial or financial relationships that could be construed as a potential conflict of interest.

## Footnotes

^1^https://db.humanconnectome.org

^2^https://pitt.box.com/v/HCP1021-1mm

^3^http://dsi-studio.labsolver.org/dsi-studio-download

^4^http://atlas.brainnetome.org/download.html
